# A spatio-temporal mining approach towards summarizing and analyzing protein folding trajectories

**DOI:** 10.1186/1748-7188-2-3

**Published:** 2007-04-04

**Authors:** Hui Yang, Srinivasan Parthasarathy, Duygu Ucar

**Affiliations:** 1 Department of Computer Science, San Francisco State University, 1600 Holloway Avenue, San Francisco, California, USA; 2 Department of Computer Science and Engineering, Ohio State University, 2015 Neil Avenue, Columbus, Ohio, USA

## Abstract

Understanding the protein folding mechanism remains a grand challenge in structural biology. In the past several years, computational theories in molecular dynamics have been employed to shed light on the folding process. Coupled with high computing power and large scale storage, researchers now can computationally simulate the protein folding process in atomistic details at femtosecond temporal resolution. Such simulation often produces a large number of folding trajectories, each consisting of a series of 3D conformations of the protein under study. As a result, effectively managing and analyzing such trajectories is becoming increasingly important.

In this article, we present a spatio-temporal mining approach to analyze protein folding trajectories. It exploits the simplicity of contact maps, while also integrating 3D structural information in the analysis. It characterizes the dynamic folding process by first identifying spatio-temporal association patterns in contact maps, then studying how such patterns evolve along a folding trajectory. We demonstrate that such patterns can be leveraged to summarize folding trajectories, and to facilitate the detection and ordering of important folding events along a folding path. We also show that such patterns can be used to identify a consensus partial folding pathway across multiple folding trajectories. Furthermore, we argue that such patterns can capture both local and global structural topology in a 3D protein conformation, thereby facilitating effective structural comparison amongst conformations.

We apply this approach to analyze the folding trajectories of two small synthetic proteins-BBA5 and GSGS (or Beta3S). We show that this approach is promising towards addressing the above issues, namely, folding trajectory summarization, folding events detection and ordering, and consensus partial folding pathway identification across trajectories.

## 1 Background

The three dimensional (3D) native structures of proteins have important implications in proteomics. Understanding such structures enables us to explore the function of a protein, explain substrate and ligand binding, perform realistic drug design and potentially cure diseases caused by protein misfolding. The protein folding problem is therefore one of the most fundamental yet unsolved problems in computational molecular biology. One major challenge in simulating the protein folding process is its complexity. Snow *et al*. state that performing a Molecular Dynamics (MD) simulation on a mini-protein for just 10 *μ*s would require decades of computation time on a typical CPU [1]. Researchers in the Folding@home project recently proposed a World Wide Web-based computing model to simulate the protein folding process [2].

As the volume of folding trajectories produced from high-throughput simulation tools increases drastically, there is an urgent need to compare, analyze, and manage such data. Previously, researchers have examined several summary statistics (e.g. radius of gyration, root mean square deviation (RMSD)) to identify similar 3D conformations in folding trajectories. Although summary statistics are commonly used for comparison, they can only capture biased and limited global properties of the conformation. Recently, Russel *et al*. [3] suggested using geometric spanners for mapping a simulation to a more discrete combinatorial representation. They apply geometric spanners to discover the proximity between different segments of a protein across a range of scales, and track the changes of such proximity over time.

To overcome the difficulties in managing and analyzing the large amount of protein folding simulation data, Berrar *et al*. [4] proposed using a data warehouse system. They embed the warehouse in a grid computing environment to enable data sharing. They also propose implementing a set of data mining algorithms to facilitate commonly needed data analysis tasks.

In this article, we propose a spatio-temporal mining approach to analyze folding trajectories. We extend the spatio-temporal data mining framework that we have developed earlier to analyze and manage such data [5]. This framework is designed to analyze spatio-temporal data produced in several scientific domains. Previously, we have applied this framework to analyze 8732 proteins taken from the Protein Data Bank to identify structural fingerprints for different protein classes (e.g., *α*-proteins) [6]. Each protein is associated with a set of objects that are extracted from its contact map. We then realize the notion of Spatial Object Association Pattern (SOAP) to effectively capture spatial relationships among such objects, Furthermore, by associating SOAPs with proteins in different protein classes, we have identified multiple types of SOAPs that can potentially function as the structural fingerprints for different protein classes. In this article, we extend such strategies to a new application domain: analyzing and characterizing the folding process of a protein.

Clearly, protein folding trajectories consist of both spatial and temporal components. Each protein in a MD simulation is composed of a number of residues spatially located in the 3D space that move over time. Each frame (or snapshot) of the trajectory can be represented as a 2D contact map, which captures the pair-wise 3D distances between residues. We extract non-local bit-patterns from these contact maps. We then use an entropy-based clustering algorithm to cluster such bit-patterns into groups. These bit-patterns are further associated to form spatial object association patterns (SOAPs). By using SOAPs, we are able to effectively summarize and analyze folding trajectories produced by MD simulations. A major advantage of this representation is its appropriateness for cross-comparison across different simulations, as discussed in later sections.

Compared to our previous work on protein structural analysis [6,7], we have made the following contributions:

• *Propose a contact map-based approach to analyze protein folding trajectories: *Our previous work focused on identifying structural signatures in native conformation of proteins in different classes or folds. Thus, there is no temporal component involved. In contrast, a folding trajectory has both spatial and temporal components. In addition, bit-patterns in a folding trajectory will interact with each other and evolve over time. Moreover, the proposed approach also effectively integrates 3D structural information in the overall analysis. This is critical in understanding the protein folding mechanism.

• *Map 2D bit-patterns in contact maps with 3D structural motifs: *To better understand and explain the biological meaning of the bit-patterns in contact maps, we have made an effort to establish a mapping between such bit-patterns and well-known structural motifs (e.g., *α*-helices and *β*-turns) in 3D conformations. Currently, this task is carried out manually. We are in the process of automating this mapping. Such a 2D-3D mapping is essential to folding data analysis due to the following reasons: First, to gain insight into the folding process, it is critical to identify the formation of important local 3D motifs such as *β*-turns. Second, our previous studies show that by associating multiple bit-patterns in contact maps, one can construct effective structural signatures for different protein classes or folds [6]. This leads us to hypothesize that a mapping might exist between 2D bit-patterns in contact maps and 3D local motifs of a protein. In this work, we validate this hypothesis and report the mapping result later. Finally, such a mapping not only enables one to take advantage of the simplicity of working in the 2D space of contact maps, but also allows one to relate to the 3D space of protein conformations. This is important in understanding the protein folding process.

• *Indirectly capture interactions among structural motifs in 3D space: *In our previous work, two bit-patterns are considered spatially proximate if they are located in the same vicinity within a 2D contact map. This is problematic in the context of protein folding, as two bit-patterns can be spatially proximate in a contact map even though their corresponding motifs are distant in the 3D conformation. (See Section 3 for more details.) We address this issue by considering the 3D distance between two bit-patterns.

• *Propose novel strategies to analyze protein folding trajectories: *We propose several novel strategies to analyze protein folding trajectories based on spatial and spatio-temporal association patterns.

In summary, one can benefit from our mining approach in two main aspects:

• **Effective, informative and scalable representation of folding simulations**: We represent each frame by a set of SOAPs, where each SOAP in turn characterizes the spatial relationship (or interactions in the folding case) among multiple bit-patterns. SOAPs are not only easily obtainable but also, as we will show, able to capture folding events along a folding trajectory.

• **Cross-analysis of trajectories to reveal a consensus partial folding pathway**: By representing each frame as a set of SOAPs, one can carry out analysis across different trajectories. Such analysis includes detecting critical events and identifying consensus partial folding pathways across trajectories.

The remainder of the article is organized as follows. In Section 2, we describe the two proteins-BBA5 and GSGS-and their trajectories produced from computational simulation. We also identify two main goals to analyze such trajectories. In Section 3, we present a step-by-step description of our analysis approach. We next report the empirical results on analyzing the trajectories of the two proteins in Section 4. We focus on the protein BBA5. Finally we conclude and report several ongoing research directions in Section 5.

## 2 Analyzing Protein Folding Trajectories

### 2.1 Protein Folding Trajectories

Advances in high-performance computing technologies and molecular dynamics have led to successful simulations of folding dynamics for (small) proteins at the atomistic level [8]. Such simulations result in a large number of *folding trajectories*, each of which consists of a series of 3D conformations of the protein under simulation. These conformations are usually sampled regularly (e.g., every 200fs) during a simulation. In this article, we also refer to each conformation as a *folding frame *or simply a *frame*. Furthermore, to represent a protein conformation, we adopt one of the commonly adopted representation schemes, where a conformation is represented as a sequence of *α*-carbons (*C*_*α*_) located in 3D space.

In this article, we focus on the folding trajectories of two mini proteins: BBA5 (Protein Data Bank ID) [9] and GSGS (orBeta3s) [10,11]. Such trajectories were produced by the Folding@ home research group at Stanford University [12].

**Figure 1:**
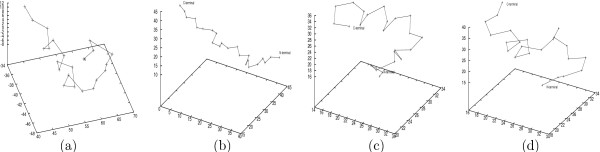
**Different conformations of the small protein BBA5, where only the *C*_*α *_atoms are shown**. (a)The native NMR structure of BBA5 based on data from the SCOP website. (b)The initial conformation of both folding trajectories. (c)The last conformation in the first trajectory. (d)The last conformation in the second trajectory.

**Figure 2:**
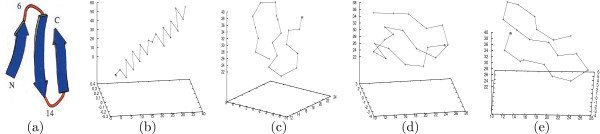
**Different conformations of the GSGS peptide, where only the *C*_*α *_atoms are shown**. (a)The native NMR conformation of GSGS. (b)The initial conformation in all the five folding trajectories. (c)The last conformation in the 1^*st *^trajectory. (d)The last conformation in the 3^*rd *^trajectory. (e)The last conformation in the 5^*th *^trajectory.

BBA5 is a 23-residue protein that folds at microsecond timescale. The native structure (or fold) of BBA5 shows a *β*-hairpin involving residues 1–10 and centering about residues 4–5. It also includes an *α*-helix involving the remaining residues 11–23. By convention, residues are numbered increasingly from the N-terminal to C-terminal of a protein. Figure 1(a) illustrates the native conformation of BBA5. The two folding trajectories, referred to as *T*_23 _and *T*_24 _respectively, are of different length. *T*_23 _consists of a series of 192 conformations (or frames), while *T*_24 _has 150 frames. Each conformation is described at atomistic level in PDB format adopted by the Protein Data Bank programs. GSGS (or Beta3s) is a 20-residue peptide with an average folding rate of microseconds. Its NMR conformation shows a three-stranded anti-parallel *β*-sheet with turns at residues 6 – 7 and 14 – 15. Figure 2(a) depicts this native conformation. There are a total of 5 GSGS folding trajectories: *T*_1_, *T*_2_, *T*_3_, *T*_4_, and *T*_5_. The number of conformations in each trajectory is listed in Table 1. Similar to BBA5, each conformation corresponds to one PDB file. Pande *et al*. explained in detail on the simulation model and methods employed to produce such trajectories [8,9].

**Table 1:** A brief description of the GSGS folding trajectories.

Trajectory ID	Total number of conformations
*T*_1_	25,664
*T*_2_	30,075
*T*_3_	19,649
*T*_4_	25,263
*T*_5_	25,664

### 2.2 Comparing Conformations of BBA5 and GSGS Across Trajectories

Although both trajectories of BBA5 start from the same extended conformation as shown in Figure 1(b), when we examine the visualized frames, they seem to identify two very different folding processes. Figures 1(c) and 1(d) illustrate the last frame in the two trajectories *T*_23 _and *T*_24 _respectively. This also applies to the five GSGS folding trajectories, where each starts with the same conformation (Figure 2(b)) but ends at a different conformation (Figures 2(c), 2(d) &2(e)).

This seeming difference might be attributed to the stochastic nature of the folding simulation process [8,9]. However, it is also desirable to characterize the similarities (or dissimilarities) across multiple trajectories.

To compare two trajectories, one must address the following key issue: how can we compare two protein conformations? Several measures have been commonly used towards such a purpose, including RMSD (root mean squared distance) [13], contact order [14], and native contacts [15]. However, all these measures are designed to quantify the global topology of a conformation. Furthermore, based on our empirical analysis of these measures, we notice that they are generally too coarse and thus can often be misleading. Even more importantly, such measures fail to identify similar local structures (or motifs) between conformations. This is especially crucial for small proteins like BBA5. As demonstrated in both experimental and theoretical studies, small proteins often fold hierarchically and begin locally [16]. For instance, it has been shown that BBA5 tends to first form secondary structures such as *β*-turns and *α*-helices, then conform to its global topology [9]. Finally, as suggested by Pande [8], both sterics (local motifs) and global topology might play an important role in protein folding. Therefore, to compare conformations of (small) proteins, a more reasonable comparison should consider both local and global structures. Moreover, it should also take the native topology of the protein under study into account.

**Figure 3:**
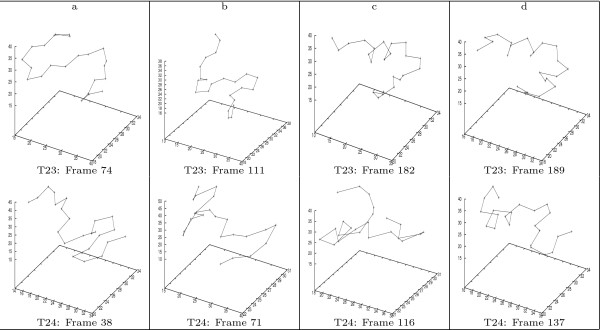
**Selected conformation-pairs along the consensus partial folding pathway of BBA5**. The figure illustrates four conformation-pairs, one from each trajectory, along the consensus partial folding pathway identified in the two BBA5 trajectories.

To meet these requirements, we propose the following two-step approach to compare conformations of BBA5. First, we partition the 23 residues of BBA5 into four fragments: (i) *F*_1_: N-terminal 1–10 *β*-hairpin; (ii) *F*_2_: C-terminal 11 – 23 *α*-helix fragment; (iii) *F*_3_: the first half of *F*_1 _and the second half of *F*_2_; and (iv) *F*_4_: the second half of *F*_1 _and the first half of *F*_2_, i.e., the middle section in the primary sequence. This segmentation of is also summarized in Table 2. Second, we recognize the secondary structure propensity in each fragment. Two conformations are said to be similar if they demonstrate the same secondary structure propensity in the same fragment. For instance, the pair of conformations in Figure 3(a) are similar as residues in *F*_1_, *F*_2 _and *F*_4 _from both conformations indicate a *β*-turn like local motif. Please note that the orientation of local motifs does not affect the comparison. For instance, in Figure 3(d), we say the two conformations have a similar structure in *F*_1 _fragment, even though the *β*-turn motifs have different orientations.

**Table 2:** Partitions along the primary sequence of BBA5.

Partition	Amino Acids	Remark
*F*_1_	1–10	*β*-hairpin
*F*_2_	11–23	*α*-helix
*F*_3_	1–6, 16–23	The 1^*st *^half of *F*_1 _and the 2^*nd *^half of *F*_2_
*F*_4_	6–17	The 2^*nd *^half of *F*_1 _and the 1^*st *^half of *F*_2_

The same two-step approach is also applied to find similar GSGS conformations, except that a different segmentation strategy is adopted according to the native GSGS structure. A total of seven segments are being used to identify the relative location of a motif in GSGS. Table 3 lists such segments. Also listed are the residues involved in each segment and its biological meaning.

**Table 3:** Partitions along the primary sequence of GSGS.

Partition ID	Amino Acids	Remark
*F*_1_	1–15	The 1^*st *^*β*-turn
*F*_2_	1–7	The 1^*st *^*β*-strand
*F*_3_	3–10	Critical region of the 1^*st *^*β*-turn
*F*_4_	6–15	The 2^*nd *^*β*-strand
*F*_5_	6–20	The 2^*nd *^*β*-turn
*F*_6_	10–18	Critical region of the 2^*nd *^*β*-turn
*F*_7_	14–20	The 3^*rd *^*β*-strand

To realize the comparison of conformations, two more issues must still be addressed. First, how can one effectively capture and represent local motifs? Second, how can we represent the global topology of a conformation in terms of local motifs? To address the first issue, we leverage the non-local patterns in protein contact maps. For the second, we characterize the spatial arrangement among non-local patterns. Please see Section 3 for more details.

### 2.3 Folding Trajectory Analysis: Objectives

There are two main goals we would like to achieve in analyzing the folding trajectories. First, we would like to address the following issues for individual trajectories: (1) to detect (or predict) significant folding events, including the formation of *β*-turns, *α*-helices, and native-like conformations; and (2) to recognize the temporal ordering of important folding events in the trajectory. For instance, between the two secondary structures *α*-helix and *β*-hairpin in BBA5, which forms earlier? What is ordering of the two events preceding a *β*-hairpin formation: formation of two extended strands or formation of the turn?

In contrast to the first goal, our second goal concerns multiple trajectories. Specifically, we would like to identify a sub-sequence of similar conformations across trajectories. This sub-sequence of conformations is referred to as the *consensus partial folding pathway*. This is analogous to the Longest Common Sub-sequence (LCS) problem [17], but much more challenging due to the following reasons. First, we are dealing with time series of 3D protein structures. Second, we are looking for *similar conformations across trajectories*, and our work on mining spatio-temporal data [5].

## 3 Algorithm

**Figure 4:**
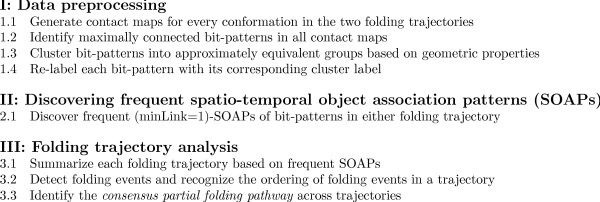
**Algorithm**. Main steps of summarizing and analyzing protein folding trajectories.

In this section, we describe in detail the proposed approach for analyzing protein folding trajectories. As shown in Figure 4, such an approach consists of three main phases: (I) Data preprocessing, (II) Spatio-temporal object association pattern mining, and (III) Trajectory analysis. We next discuss each phase in further details.

### 3.1 Data Preprocessing

Same as in our previous studies on protein structural analysis [6,7], we represent 3D protein conformations by contact maps. In order for this algorithm to be self-contained, we next briefly go over these preprocessing steps. We also explain the rationale of such steps in the context of protein folding.

#### Contact Map Generation

When generating contact maps, we consider the Euclidean distances between *α*-carbons (*C*_*α*_) of each amino acid. Two *α*-carbons are considered to be in contact if their distance is within 8.5 Å. Thus, for a protein of *N *residues, its *contact map *is an *N *× *N *binary matrix, where the cell at (*i*, *j*) is 1 if the *i*^*th *^and *j*^*th *^*α*-carbons are in contact, 0 otherwise. Since contact maps are symmetric across the diagonal, we only consider the bits below the diagonal. Furthermore, we also ignore the pairs of *C*_*α *_atoms whose distance in the primary sequence is ≤ 2, as they are sure to be in contact. This step transforms the two BBA5 trajectories into two series of contact maps, with each contact map of size 23 × 23. By the same token, the 5 GSGS trajectories are transformed into 5 sequences of contact maps.

#### Identifying Maximally Connected Bit-patterns

Every bit in a contact map has eight neighbor bits. For an edge position, we assume its out-of-boundary positions contain 0. In a contact map, a connected bit-pattern is a collection of bit-1 positions, where for each 1, at least one of its neighbors is 1. Correspondingly, we define a *maximally-connected bit-pattern *(also referred to as a *bit-pattern *in this article) to be a connected pattern *p *where every neighbor bit not in *p *is 0. We apply a simple region growth algorithm to identify all the *maximally-connected patterns *in each contact map within the two series of contact maps, corresponding to the two folding trajectories of BBA5. Altogether, we identified 352 maximally-connected bit-patterns in such contact maps. For the GSGS folding data, a total of 50,572 unique bit-patterns are constructed. We then represent each identified bit-pattern as a 6-tuple feature vector consisting of the following attributes:

• *Height*: the number of rows contained in the pattern's Minimum Bounding Rectangle (MBR).

• *Width*: the number of columns in the pattern's MBR.

• *NumOnes*: the number of 1s in the pattern.

• *Slope*: the general linear distribution trend of all the 1s in the pattern within its MBR. To compute the angle of a connected pattern we use the least-squares method to estimate the slope of a linear regression line. For a pattern containing *n *1s, we denote the positions of the 1s as: (*x*_1_, *y*_1_)...(*x*_*n*_, *y*_*n*_). The least-squares method then estimates the slope *β*_1 _as: β1=∑i=1n((xi−x¯)∗(yi−y¯))/∑i=1n((xi−x¯)2)
 MathType@MTEF@5@5@+=feaafiart1ev1aaatCvAUfKttLearuWrP9MDH5MBPbIqV92AaeXatLxBI9gBaebbnrfifHhDYfgasaacH8akY=wiFfYdH8Gipec8Eeeu0xXdbba9frFj0=OqFfea0dXdd9vqai=hGuQ8kuc9pgc9s8qqaq=dirpe0xb9q8qiLsFr0=vr0=vr0dc8meaabaqaciaacaGaaeqabaqabeGadaaakeaaiiGacqWFYoGydaWgaaWcbaGaeGymaedabeaakiabg2da9maaqadabaGaeiikaGIaeiikaGIaemiEaG3aaSbaaSqaaiabdMgaPbqabaGccqGHsislcuWG4baEgaqeaiabcMcaPiabgEHiQiabcIcaOiabdMha5naaBaaaleaacqWGPbqAaeqaaOGaeyOeI0IafmyEaKNbaebacqGGPaqkcqGGPaqkcqGGVaWldaaeWaqaaiabcIcaOiabcIcaOiabdIha4naaBaaaleaacqWGPbqAaeqaaOGaeyOeI0IafmiEaGNbaebacqGGPaqkdaahaaWcbeqaaiabikdaYaaakiabcMcaPaWcbaGaemyAaKMaeyypa0JaeGymaedabaGaemOBa4ganiabggHiLdaaleaacqWGPbqAcqGH9aqpcqaIXaqmaeaacqWGUbGBa0GaeyyeIuoaaaa@5A04@

• *xStdDev*: the standard deviation of all the 1s' x-coordinates (this quantifies how the 1s spread along the x dimension).

• *yStdDev*: the standard deviation of all the 1s' y-coordinates.

Note that this feature vector captures the main geometric properties of a bit-pattern.

As discussed in the literature [18-21], non-local patterns (where bit-patterns are one type of non-local patterns,) in contact maps can effectively capture the secondary structure of proteins. Our previous work [6,7] demonstrated that by characterizing the spatial relationship among the above described bit-patterns, one can construct structural signatures for proteins of different classes or folds. In the context of protein folding, we have observed that the above-defined bit-patterns are also capable of capturing a wide range of local 3D structural motifs. They can even approximately measure the strength of secondary structure propensity in a conformation. For instance, we have identified bit-patterns that correspond to "premature" *α*-helices and native-like *α*-helices respectively. Henceforth, we refer to the 3D structure formed by all the participating residues of a bit-pattern as the *3D motif of the bit-pattern*. The relationship between bit-patterns and 3D motifs will be further discussed in the next section.

#### Clustering Bit-patterns into Approximately Equivalent Groups

In this step, we partition the extracted bit-patterns into *approximately equivalent groups*, each of which consists of bit-patterns that exhibit similar geometric properties (e.g., shape and size). To construct such equivalent groups, we run the *k*-means based clustering algorithm [22] over the bit-patterns' corresponding feature vectors, where *k *is the number of clusters (or equivalent groups) that will be produced.

To determine an optimal value of *k*, we take the following three steps. First, we run the clustering algorithm on different *k *values. This produces different clustering schemes for the same set of bit-patterns. Second, for each clustering scheme, we compute its entropy. Let *c*_1_, ..., *c*_*l *_be the *l *clusters after clustering the set of *N *bit-patterns. Furthermore, each cluster *c*_*i *_(1 ≤ *i *≤ *l*) has an individual entropy *H*_*i *_and contains *N*_*i *_elements, then the total entropy of this clustering is given by the following formula: H=∑i=1kHi∗(Ni/N)
 MathType@MTEF@5@5@+=feaafiart1ev1aaatCvAUfKttLearuWrP9MDH5MBPbIqV92AaeXatLxBI9gBaebbnrfifHhDYfgasaacH8akY=wiFfYdH8Gipec8Eeeu0xXdbba9frFj0=OqFfea0dXdd9vqai=hGuQ8kuc9pgc9s8qqaq=dirpe0xb9q8qiLsFr0=vr0=vr0dc8meaabaqaciaacaGaaeqabaqabeGadaaakeaacqWGibascqGH9aqpdaaeWaqaaiabdIeainaaBaaaleaacqWGPbqAaeqaaOGaey4fIOIaeiikaGIaemOta40aaSbaaSqaaiabdMgaPbqabaGccqGGVaWlcqWGobGtcqGGPaqkaSqaaiabdMgaPjabg2da9iabigdaXaqaaiabdUgaRbqdcqGHris5aaaa@3F89@ The entropy of each individual cluster, i.e., *H*_*i *_, is computed by summing up the entropy of each of the six bit-pattern attributes such as its height and width. For an attribute, we compute its entropy in a cluster according to the procedure explained by Shannon [23]. In the third and final step, we plot the entropy against the number of clusters, i.e., *k*, and choose a value *k *where the entropy plot begins to show a linear trend. For the BBA5 folding data, this clustering step groups the 352 bit-patterns into 10 clusters (or types). As for the GSGS data, 12 clusters are identified.

**Figure 5:**
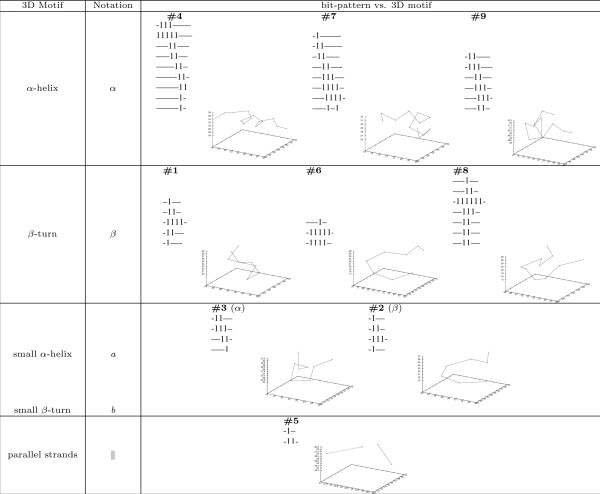
**Mapping between 2D bit-patterns and 3D sub-structures**. The figure visualizes the representative 3D sub-structures corresponding to the 10 classes of bit-patterns identified in the contact maps along BBA5's two folding trajectories. The bit-patterns shown here are randomly selected from their respective group for illustration purpose.

Intuitively, the 3D motifs of the bit-patterns in a cluster will also have similar 3D geometric properties. This is verified based on our manual analysis on the BBA5 trajectories. Figure 5 illustrates the representative 3D motifs.corresponding to the 9 of 10 types of bit-patterns identified in BBA5 trajectories. We omit type 0, as bit-patterns of this type, unlike the others, correspond to a wide variety of 3D motifs.

We also observed a similar scenario for the 12 types of bit-patterns identified in the GSGS trajectories. For instance, the typical 3D motifs of type 0 bit-patterns resemble the native conformation of GSGS (See Figure 2(a)); whereas those of type 6 identify with *α*-helices.

**Figure 6:**
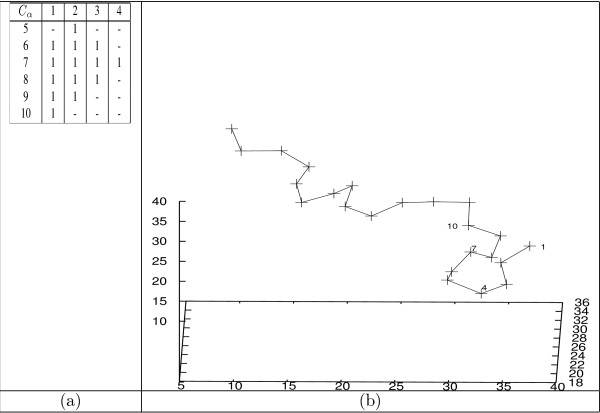
***β*-turns vs. maximally connected bit-patterns: an example**. (a) A type 8 bit-pattern is identified in the 166^*th *^frame of the BBA5 *T*23 trajectory. This bit-pattern corresponds to the the connected 1s in the table, where a '1' indicates two corresponding *C*_*α *_atoms are in contact,'-' otherwise. This pattern consists of the first 10 *C*_*α *_atoms. (b) The 3D conformation of this frame, where the first 10 *C*_*α *_atoms resembles a *β*-turn.

**Figure 7:**
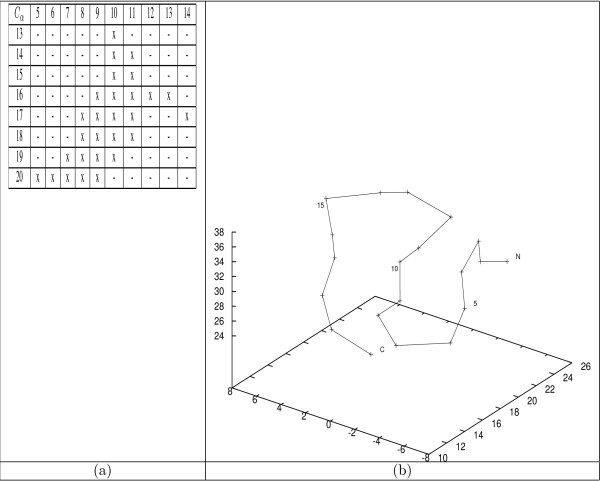
***β*-sheets vs. maximally connected bit-patterns: an example**. (a) A type 0 bit-pattern is identified in the 24201^*th *^frame of the GSGS *T*_1 _trajectory. This bit-pattern corresponds to the the connected 'x'-es in the table, where an 'x' indicates two corresponding *C*_*α *_atoms are in contact,'-' otherwise. It consists of *C*_*α *_atoms from 5 through 20. (b) The 3D conformation of this frame, where the 5–20 *C*_*α *_atoms resembles a *β*-sheet of two turns.

Upon a closer look at this 2D-3D mapping illustrated in Figure 5, one can observe the following interesting aspects. First, multiple types of bit-patterns can be associated with a single type of 3D motif. For instance, there are 3 types of bit-patterns are mapped to an *α*-helical motif. Second, contrary to a commonly accepted belief that *β*-turns or *β*-sheets cannot be captured by maximally connected bit-patterns as defined earlier, our analysis shows that this belief does not stand. To illustrate this point, we take two examples. The first example, illustrated in Figure 6, corresponds to the *β*-turn structure. As shown in Figure 6(b), the *β*-turn formed by the first 10 *C*_*α *_atoms of BBA5 can be captured by the maximally connected bit-pattern shown in Figure 6(a). The second example, shown in Figure 7, illustrates that a two turn *β*-sheet (Figure 7(b)) can also be captured by a bit-pattern (Figure 7(a)). Finally, not every type of bit-patterns can be mapped to a typical 3D motif. This might be attributed to our entropy-based criteria for selecting an "optimal" value of the parameter *k *in the clustering task.

This demonstrates, to a certain extent, the advantage of using 2D contact maps to analyze 3D protein conformations. Undoubtedly, using contact maps greatly reduces the computational complexity, though at the cost of loss in structural information. However, some of this information loss is re-compensated by mapping bit-patterns to structural motifs in 3D conformations. More importantly, by exploiting different features in contact maps (bit-patterns in this work), we are able to connect 2D features with features in 3D space. In the BBA5 case, by identifying 10 types of bit-patterns in contact maps, we indirectly recognize 10 different 3D structural motifs in the folding conformations.

#### Re-labeling Bit-patterns with The Corresponding Cluster Label

In this step, we re-label all the previously identified bit-patterns with their corresponding cluster label. Let *p *be a labeled bit-pattern. It can be represented as follows: *p *= (*trajID*, *frameID*, *listC*_*α*_, *label*). Here, *trajID *identifies a folding trajectory, and *frameID *indicates the frame where *p *occurs, *listC*_*α *_consists of all participating *α*-carbons of *p*, identified by their position in the primary sequence. Finally, *label *is the cluster label of *p*. For BBA5, *label *∈ {*g*_0_, *g*_1_, ⋯, *g*_9_}, corresponding to the 10 approximately equivalent groups (or types).

### 3.2 Mining Spatio-temporal Object Association Patterns

The preprocessing steps transform a 3D protein conformation into a set of labeled 2D bit-patterns, that indirectly capture the local 3D structural characteristics of the conformation. For the two BBA5 trajectories, each conformation contains an average of 6 bit-patterns. As for the five GSGS trajectories, the average number of bit-patterns in each conformation is 4.

As BBA5 and GSGS fold, the dynamics among their residues is constantly changing until it reaches an equilibrium. This means that two residues previously in contact may become out of contact later. As a result, bit-patterns present in one conformation may be absent in the next. The evolving nature of contacting residues and in turn bit-patterns, is essentially the consequence of a variety of weak interactions among amino acids at different levels. Such weak interactions include hydrogen bonds, electrostatic interactions, van der Waal's packing and hydrophobic interactions [24]. To capture these (potential) interactions, a simple yet effective method is to consider how close two amino acids are located from each other in 3D. We also adopt this method here. Specifically, we consider interactions between local 3D motifs captured by labeled bit-patterns. We denote such interactions as "interactions among bit-patterns". Let *p*_*i *_and *p*_*j *_be two bit-patterns in a protein conformation, and *p_*i*_.listC*_*α *_and *p_*j*_.listC*_*α *_be the list of *α*-carbons involved in *p*_*i *_and *p*_*j*_, respectively. We define *p*_*i*_and *p*_*j *_as *interacting bit-patterns *if at least one pair of *α*-carbons, each from *p_*i*_.listC*_*α *_and *p_*j*_.listC*_*α *_are located within a short distance *δ*. Note that the value of *δ *should be greater than the distance that is being used to identify contacting *α*-carbons when generating contact maps. In our analysis, we set *δ *= 10 Å.

**Figure 8:**
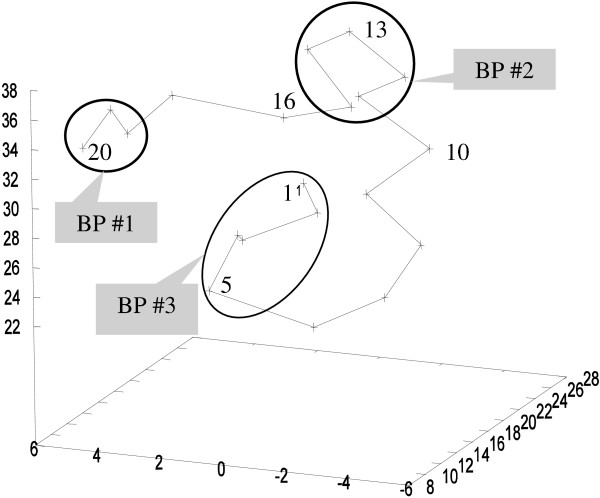
**Discrepancy between distances in 2D and 3D spaces**. Bit-patterns that are close to each other in the 2D contact map space, for instance, BP#1 and BP#2, can be distant from each other in 3D. Similarly, bit-patterns that are distant in 2D space, for instance, BP#1 and BP#3, can be close to each other in 3D.

It is noteworthy that the above notion of interacting bit-patterns is new compared to our previous work, where two bit-patterns are associated if their distance in the 2D contact map space is below a certain threshold. This can be misleading in the context of protein folding analysis. As demonstrated in Figure 8, the two bit-patterns-*BP #1 *and *BP *#*2*-are only 2 amino acids away in the 2D contact map. However, they can be relatively far apart in 3D. On the other hand, although the bit-patterns *BP #2 *and *BP #3 *are relatively far apart from each other in the 2D contact map, they are close to each other in 3D. Therefore, measuring the distance between bit-patterns in the actual 3D conformation is more robust with respect to capturing potential interaction among local motifs.

So far, we have discussed our approach of using bit-patterns in contact maps to characterize local 3D motifs and further represent a protein conformation during folding. We also define the notion of interacting bit-patterns in the folding context. We are now ready to present our method of summarizing folding trajectories to fulfill the two objectives described in Section 2.3. The main idea is that we can summarize a folding trajectory by characterizing the evolutionary behavior of interactions among different types of bit-patterns and in turn, the interactions among local 3D motifs.

#### Definition of (minLink = 1) SOAP

As proposed in our previous work [5,25], such interactions can be modeled and captured by discovering different types of spatial object association patterns (SOAPs). Essentially, SOAPs characterize the specific way that objects, bit-patterns in this case, are interacting with each other at a given time. Among the proposed SOAP types, after a careful evaluation, we empirically select (*minLink *= 1) SOAPs to model the interacting bit-patterns in the folding process. Let *p *= (*g*_1_, *g*_2_, ⋯, *g*_*k*_) be a (*minLink *= 1) SOAP of size *k*, where *g*_*i *_is one of the 10 types of bit-patterns described above. In the context of folding trajectories, *p *prescribes that there exists *k *bit-patterns *b*_1_, *b*_2_, ..., *b*_*k *_in a conformation, where *b*_*i*_.*label *= *g*_*i *_(1 ≤ *i *≤ *k*). Furthermore, for each *b*_*i*_, it interacts with at least one of the remaining (*k *- 1) bit-patterns. Note that the *k *labels in *p *are not mutually exclusive. For instance, one can have SOAPs such as (7 9 9), which involves one type 7 bit-pattern and two type 9 bit-patterns.

We further restrict ourselves to SOAPs that occur frequently during the folding process (*frequent SOAPs*). However, we are not ruling out rarely-occurring SOAPS in our future studies. A SOAP is said to be frequent if it appears in no fewer than *minSupp *frames in a trajectory. In our studies, we set *minSupp *= 5 for BBA5 and 10 from GSGS.

#### SOAP Episodes

The next step is to capture the evolutionary nature of the folding process. We do this by identifying the evolutionary nature of SOAPs. As mentioned earlier, small proteins like BBA5 and GSGS often fold hierarchically and begin with local folded structures. As they fold, new SOAPs can be created and existing one can dissipate. To capture such evolutionary behavior, we proposed the concept of *SOAP episodes*, which provide an effective approach to model the evolution of interactions among spatial objects over time [5]. To reiterate, a SOAP episode *E *is defined as follows: *E *= (*p*, *F*_*beg*_, *F*_*end*_), where *p *is a SOAP composed of one or more bit-patterns, *p *was created in frame *F*_*beg *_and persisted till frame *F*_*end*_. Note that for a given *p*, it can be created more than once during protein folding, and thus can have more than one episode. To discover frequent (*minLink *= 1) SOAPs and their episodes in the trajectories of BBA5 and GSGS, we apply our SOAP mining algorithm as explained in our previous work [5].

In summary, this mining phase produces the following results: (i) A list of (*minLink *= 1) SOAPs of bit-patterns that appeared in at least 5 conformations in each folding trajectories for the protein BBA5 and 10 for GSGS; and (ii) A list of episodes, ordered by beginning frame *F*_*beg*_, associated with each of these SOAPs.

### 3.3 Folding Trajectory Analysis

In this section, we describe our strategy on utilizing SOAPs to summarize a folding trajectory and address the two folding analysis issues described in Section 2.3.

#### SOAP-based Trajectory Summarization

The previous mining phase discovers a collection of frequent (*minLink *= 1) SOAPs and the associated episodes in each trajectory. Therefore, it identifies all the conformations in the trajectories that contain at least one frequent (*minLink *= 1) SOAPs. For instance, the last conformation in trajectory *T*23 (Figure 1(c)) has two SOAPs of size 2:(5 8) (i.e., association of a type 5 and a type 8 bit-pattern) and (7 8), and three SOAPs of size 1: (5), (7), and (8), while the last conformation in trajectory *T*24 has three SOAPs: (7 8), (7) and (8). This leads to our SOAP-based approach for folding trajectory summarization.

To summarize a folding trajectory, we perform the following three steps. First, for each conformation, we identify all the frequent SOAPs that appear in it and use these SOAPs to represent this conformation. Note that not every conformation contains frequent SOAPs, especially when *minSupp *is set high. Second, for each SOAP-representable conformation, we carry out two tasks on its associated SOAPs. We next use the folding trajectories of BBA5 to explain how these two tasks are carried out.

**Figure 9:**
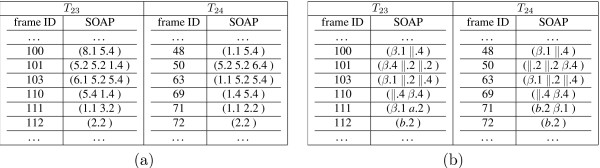
**SOAP-based folding trajectory summarization**. An sample segment in each of the two BBA5 folding trajectories is presented, (a) After superimposing the relative location of each bit-pattern and pruning away redundant SOAPs. (b) After further generalizing each bit-pattern by corresponding 3D motif.

In the first task, for each SOAP, we mark the relative location of each involved bit-pattern in the primary sequence of BBA5. This is done by identifying the segment of BBA5 where the majority of a bit-pattern's *α*-carbons are located. The segment can be one of the following as described in Section 2.2: *F*_1_, residues 1 – 10; *F*_2 _, residues 11 – 23; *F*_3_, residues 6–17; and *F*_4_: residues 1–5 and 18–23. Let us again take the last conformation in *T*24 as an example. It can be summarized by three SOAPs: (7 8), (7) and (8). When we look at the list of *α*-carbons involved in these bit-patterns, we find out that 7 is mainly located in *F*_2 _and 8 in *F*_1_. Therefore, we mark the three SOAPs as follows: (8.1 7.2), (7.2) and (8.1). We re-arrange the bit-patterns in a SOAP by their relative locations in BBA5. This super-imposes BBA5-specific spatial information to a SOAP. In the second task, we prune away redundant SOAPs after marking each bit-pattern with its relative location in BBA5. A SOAP is redundant if it is embedded in another SOAP. For instance, in the previous example, we can prune away (8.1) and (7.2) as both are embedded in (7.2 8.1). After pruning, most conformations in such a small protein can often be represented by a single SOAP. We can even take this summarization a step further, where we replace a bit-pattern with its corresponding 3D motif, as illustrated in Figure 5. For instance, SOAP (7.2 8.1) will be transformed into (*β*.1 *α*.2). We refer to such SOAPs as *generalized SOAPs*, and the corresponding trajectory as *a generalized trajectory*. Note that in a generalized trajectory, multiple types of bit-patterns can be mapped into a single type of 3D motif. For instance, the *α*-motif corresponds to three types of bit-patterns 4, 7, and 9 (Figure 5). Figure 9 shows a segment in each summarized BBA5 folding trajectory before and after being generalized with 3D motifs.

#### Detecting Folding Events and Recognizing Ordering Among Events

Once each folding trajectory is summarized into generalized SOAPs, it is fairly straightforward to detect folding events such as the formation of *α*-helix or *β*-turn like local structures. This can be done by simply locating the frames that contain the local motif(s) of interest. We can also easily identify native-like conformations, by finding those that contain the generalized SOAP (*β*.1 *α*.2). Finally, based on the summarization, one can quickly identify the ordering of folding events in a trajectory. For instance, to check which secondary structure forms more rapidly, *α*-helix or *β*-hairpin, one can simply compare the first occurrence of these structures in the summarized trajectory (Figure 9(b)).

#### Identifying the Consensus Partial Folding Pathway Across Trajectories

To do this, we simply compute the longest common sub-sequence (LCS) [17] between two summarized trajectories. One can utilize the summarization either before the 3D motif generalization (Figure 9(a)) or after (Figure 9(b)). We use the latter in our analysis. Based on the LCS of generalized SOAPs, we construct the consensus folding pathway by identifying pairs of conformations, one from each trajectory, along the LCS of two summarized trajectories. In other words, the resulting consensus pathway consists of a sequence of conformation-pairs of similar 3D structures. Notice here that the comparison between 3D protein conformations (as described in Section 2.2) is done by using bit-patterns to model local structural motifs, and associations of bit-patterns (SOAPs) to characterize the global structure. This forms a hierarchical comparison and is in accordance with the hierarchical folding process of small proteins.

## 4 Results

In this section, we report results on analyzing the two trajectories of the small synthetic protein BBA5 and the five trajectories of another small protein GSGS. However, we will focus on BBA5. In previous sections, we have described in detail the structure of BBA5 of GSGS and their folding trajectories. Such information is summarized and tabulated in Table 4 and Table 5.

**Table 4:** A summary of the BBA5 folding trajectories.

Protein	PDB Identifier: BBA5; Primary sequence: 23 residues; Designed protein; Native fold: N-terminal 1–10 *β *hairpin, C-terminal 11–23 *α*-helix
Trajectory	Two trajectories: *T*23 and *T*24; *T*23: 192 conformations; *T*24: 150 conformations
Contact map	Based on contacts between *α*-carbons. Two *α*-carbons are in contact if their Euclidian distance is ≤ 8.5 Å
Bit-patterns	A total of 352 unique maximally connected bit-patterns were identified from all conformations; Average number of bit-patterns per conformation is 6; Bit-patterns are further classified into 10 approximately equivalent types
Interacting bit-patterns	If at least one pair of *α*-carbons, one from each bit-pattern, is of Euclidian distance ≤ 10 Å
Frequent SOAPs	A SOAP is frequent if it appears in ≥ 5 conformations; A total of 444 frequent SOAPs identified in trajectory *T*23, and 258 in *T*24
Consensus partial folding pathway	We identified a consensus partial folding pathway across the two trajectories. It is composed of 71 pairs of similar conformations, one from each trajectory

**Table 5:** A summary of the GSGS folding trajectories.

Protein	Name: GSGS or Beta3s; Primary sequence: 20 residues; Designed protein; Native fold: three stranded anti-parallel *β*-sheets with turns at 6–7 and 14–15
Trajectory	Five trajectories: *T*1, *T*2, *T*3, *T*4 and *T*5; *T*1 : 25, 664 conformations; *T*2 : 30, 075 conformations; *T*3 : 19, 649 conformations; *T*4 : 25, 263 conformations; *T*5 : 25, 664 conformations;
Contact map	Based on contacts between *α*-carbons. Two *α*-carbons are in contact if their Euclidian distance is ≤ 8.5 Å
Bit-patterns	A total of 50, 572 unique maximally connected bit-patterns were identified from all conformations; Average number of bit-patterns per conformation is 4; Bit-patterns are further classified into 12 approximately equivalent types
Interacting bit-patterns	If at least one pair of *α*-carbons, one from each bit-pattern, is of Euclidian distance ≤ 10 Å
Frequent SOAPs	A SOAP is frequent if it appears in ≥ 10 conformations;

### 4.1 Detecting and Ordering Folding Events

We summarize both folding trajectories of BBA5 into a sequence of SOAPs as illustrated in Figure 9. Coincidently, both summarized trajectories consist of 64 conformations.

Based on these summarized trajectories, we can quickly identify all the conformations where the first *α*-helix-like or *β*-turn-like local motifs were formed. For trajectory *T*_23_, the first *α*-helix-like motif was identified in frame 26, and the first *β*-turn-like local motif was formed in frame 63. For the other trajectory *T*_24_, the frames were 29 and 38. This is in accordance with experimental results that *α*-helices generally fold more rapidly than *β*-turns. However, since we only consider frequent SOAPs, it is very possible that we might miss the actual first formation of such local motifs. To address this issue, we might need to consider rarely occurring SOAPs. We plan to investigate this in the future. For the two events related to *β*-turn formation, formation of two extended strands and formation of the turn, we found that for both trajectories, the formation of extended strands preceded the formation of the turn.

**Figure 10:**
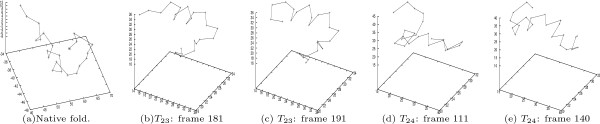
**The native-like conformations identified in the two BBA5 trajectories**. According to the SOAP-based summarization of the two BBA5 folding trajectories, two native-like conformations are identified in each trajectory.

Also, we identify two conformations in each trajectory that show native-like structure. We do this by locating the conformations associated with the generalized SOAP (*β*.1 *α*.2). Figure 10 presents the 3D structure of these native-like conformations along with the native conformation of BBA5. One can see that our SOAP-based comparison does well in identifying similar 3D conformations.

### 4.2 Consensus Partial Folding Pathway Across Trajectories

Based on the generalized trajectory summarization of BBA5, we identify a consensus partial folding pathway of length 71. In other words, 71 pairs of conformations, one from each trajectory, are considered similar to each other. Figure 3 displays four such pairs along this consensus folding pathway. For instance, the two conformations shown in Figure 3(c), corresponding to the 182^*th *^frame in the *T*_23 _trajectory and the 116^*th *^frame in the *T*_24 _trajectory of BBA5 respectively, are considered structurally similar, since both conformations exhibit an *α*-helix in the left half of the backbone, and a *β*-turn in the right half.

**Figure 11:**
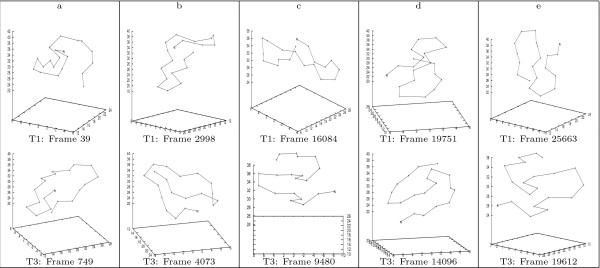
**Selected conformation-pairs along the consensus partial folding pathway across the 1^*st *^and 3^*rd *^trajectories of the GSGS peptide**. The figure illustrates five pairs of conformations, one from each trajectory, along the consensus partial folding pathway identified in the 1^*st *^and 3^*rd *^trajectories.

**Figure 12:**
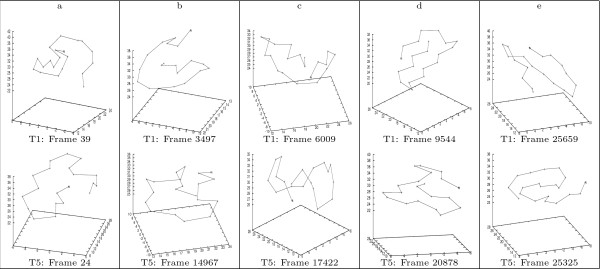
**Selected conformation-pairs along the consensus partial folding pathway across the 1^*st *^and 5^*th *^trajectories of the GSGS peptide**. The figure illustrates five pairs of conformations, one from each trajectory, along the consensus partial folding pathway identified in the 1^*st *^and 5^*th *^trajectories.

Figure 11 illustrate 5 pairs of conformations along the consensus folding pathway of the 1^*st *^and 3^*rd *^trajectories of GSGS. And Figure 12 illustrates 5 conformation-pairs along consensus pathway of the 1^*st *^and 5^*th *^trajectories of GSGS. We are currently in the process of identifying consensus pathways across more than 2 trajectories of GSGS. Note that by using bit-patterns, we naturally realize a rotation-invariant comparison. To illustrate this, let us again examine the afore-discussed conformation pair of BBA5. One notices that although the *β*-turn in the two conformations orients differently, the two conformations are still identified as being structurally similar by our approach.

Currently, we rely on visual tools to justify these consensus pathways. We did attempt to use several measurements that have been used previously to quantify the similarity between 3D protein conformations, but to no avail. These measurements include *RMSD*, contact order, and native contacts. If we identify the pathway based on the best match given by any of the above measurements, we often ended up with a very short consensus pathway (as short as 10 frames). Two conformations are said to be a best match if they have the lowest RMSD or have the smallest difference in contact order or native contacts. Moreover, different best-matched measurements rendered very different consensus pathways. Finally, we notice that the best-matched conformations based on any of such measurements can often exhibit very different structural characteristics. We are investigating alternative methods for quantitative validation of our results.

## 5 Conclusions and Ongoing Work

In this article, we present a novel approach to analyze protein folding trajectories and a case study on the small proteins BBA5 and GSGS. We capture a variety of structural motifs in the 3D protein conformations by non-local bit-patterns identified in their 2D contact maps. By modeling the interactions or spatial relationships among bit-patterns as SOAPs and SOAP episodes, we effectively characterize the evolutionary nature of the folding process. We also describe two methods to summarize folding trajectories by super-imposing protein specific information and 3D motifs onto SOAPs. Utilizing the summarized trajectories, we demonstrate that one can detect folding events and the temporal order among events. We also show that through comparing such summarized trajectories, one can identify a partial folding pathway common to multiple trajectories.

We realize that it is a very hard and challenging task to understand the folding mechanism of proteins. Based on our analysis results over a small protein, we are not in the position to make any general comments on the protein folding problem. However, the approach presented here is general and applicable to any folding trajectories.

Presently, we are in the process of addressing several other related issues. First, we are automating the mapping between 2D bit-patterns and 3D motifs. Second, we are further analyzing the identified consensus folding pathways and validating them through other means. Third, it is well-known that the side chains of a protein play a crucial role in the folding process. We are currently investigating different approaches to involve side chains in our analysis. Finally, we are investigating whether bit-patterns can be used to index and manage protein folding simulation data.
